# Measuring the Cooling Behavior of Melt Pools in L-PBF by Pyrometry

**DOI:** 10.3390/ma16103647

**Published:** 2023-05-10

**Authors:** Aron Pfaff, Sebastian Schäffer, Martin Jäcklein, Frank Balle

**Affiliations:** 1 Fraunhofer Institute for High–Speed Dynamics, Ernst-Mach-Institut, Ernst-Zermelo-Str. 4, 79104 Freiburg, Germany; sebastian.schaeffer@emi.fraunhofer.de (S.S.); martin.jaecklein@emi.fraunhofer.de (M.J.); frank.balle@mail.inatech.uni-freiburg.de (F.B.); 2 Walter-and-Ingeborg-Herrmann Chair for Power Ultrasonics and Engineering of Functional Materials (EFM), Department for Sustainable Systems Engineering (INATECH), Faculty of Engineering, University of Freiburg, 79110 Freiburg, Germany; 3 Freiburg Materials Research Center (FMF), Stefan-Meier-Str. 21, 79104 Freiburg, Germany

**Keywords:** laser powder bed fusion, pyrometry, cooling behavior, 30CrMoNb5-2, cooling duration, microstructure

## Abstract

This study aims to measure the cooling rates or, more precisely, the cooling durations of single laser tracks by pyrometry within the laser powder bed fusion (L-PBF) process. Two-color, as well as one-color pyrometers are tested within this work. Regarding the second, the emissivity of the investigated 30CrMoNb5-2 alloy is determined in-situ within the L-PBF system in order to measure temperature instead of arbitrary units. This is done by heating up printed samples and verifying the measured pyrometer signal by comparing it to values obtained by thermocouples attached to the samples. In addition, the precision of two-color pyrometry is verified for the given setup. Following the verification experiments, single laser track experiments are conducted. The obtained signals prove to be partially distorted mainly due to by-products such as smoke and weld beads arising from the melt pool. To encounter this problem, a new fitting method is presented and experimentally validated. Melt pools resulting from different cooling durations are analyzed by EBSD. These measurements show areas of extreme deformation or potential amorphization correlating with the cooling durations. The obtained cooling duration can be used for the validation of simulations as well as for the correlation of corresponding microstructure and process parameters.

## 1. Introduction

### 1.1. Motivation

Laser powder bed fusion (L-PBF) is the best-established metal additive manufacturing (AM) technology. Like most AM technologies, it is an intricate process due to the local creation of material [1]. Local imperfections or disturbances within the manufacturing process can cause defects. Therefore, a series of process monitoring technologies have been introduced. Co-axial pyrometry, as one of those, provides pointwise measurement, offering a high resolution as well as the possibility to measure the temperature within the melt pool. Thus, the technology is well suited to investigate cooling rates [2]. In general, the knowledge about cooling rates would support the validation of process simulations as well as the understanding of microstructural formation. Furthermore, the incremental nature of L-PBF offers the potential to apply locally varying process parameters in order to create functionally graded materials (FGMs), as shown by Popovich et al. [3], Pfaff et al. [4] or Kürnsteiner et al. [5]. Knowledge about the correlations between process parameters and cooling rates will support the design of further future FGMs. 

### 1.2. State of the Art

McCann et al. [2], Everton et al. [6], Hou et al. [7] as well as Yang et al. [8] provide an overview of L-PBF process monitoring technologies and their challenges. Here, McCann et al. [2] point out the main problem of pyrometry: Without knowledge about the emissivity of the regarded material, only qualitative statements can be made. However, the emissivity depends on unknown factors such as temperature, surface condition and aggregate state. Mohr et al. [9] show a first approach to how emissivity can be measured within an L-PBF system. Pavlov et al. [10] point out another challenge regarding pyrometry. Depending on the measurement setup and the chosen process parameters, the observed melt pool can be smaller than the field of measurement. For one-channel pyrometry, however, the measured temperatures depend on the average intensity measured by the infra-red sensor resulting in an underestimation of the temperature. Gutknecht et al. [11] compare co-axial two-color pyrometry with thermographic and acoustic process monitoring. The measured temperatures seem to be below the melting point of the investigated material and are therefore presented as arbitrary units. Gutknecht et al. [11] are measuring co-axially to the laser beam and, therefore, within the melt pool. The change of aggregate state is named as the reason for arbitrary units, even though two-color pyrometry is used. In the field of laser welding, however, two-color pyrometry is used for quantitative measurements within the melt pool shown in the example by Xiao et al. [12]. Alternatives to pyrometer measurements are shown, for example, by Hooper [13] and Scipioni et al. [14], who measure melt pool cooling rates with high-speed cameras. Thermography, for example, presented by Altenburg et al. [15], is a popular approach to melt pool observations. However, the resolution is limited, and the measurement setup, as well as the data analysis, are complex. Farshidianfar et al. [16] use thermography in order to link the cooling rates with grain size, phase transformations and hardness. In addition, Takata et al. [17] link the cooling rates and resulting microstructures. Pyrometry measurements have not been linked yet with microstructural analysis. Based on Hooper [13], Scipioni et al. [14] and Farshidianfar et al. [16], cooling rates in the order of 10^1^–10^7^ K/s can be expected within L-PBF.

The literature describes pyrometry as a promising, affordable and robust candidate for melt pool recording as well as process monitoring in L-PBF (e.g., see McCann et al. [2], Everton et al. [6], Yang et al. [8], Gutknecht et al. [11] or Hooper [13]). Since most studies focus on the potential of using pyrometry for processing and quality monitoring (e.g., see [7,18,19,20]), the obtained signals are mainly interpreted as arbitrary units. Measuring temperatures instead of arbitrary units remains challenging due to the dependency of the emissivity from unknown factors such as temperature, surface condition and aggregate state. 

### 1.3. Objective

The main goal of the presented research paper is to measure quantitative cooling rates by pyrometry. Therefore, an approach to measure quantitative temperatures within L-PBF instead of arbitrary units is introduced. Systematic single laser track experiments regarding the impact of laser power, scanning speed and laser focus diameter are combined with EBSD measurements in order to correlate the microstructures and cooling durations. 

## 2. Materials and Methods

**Figure 1:**
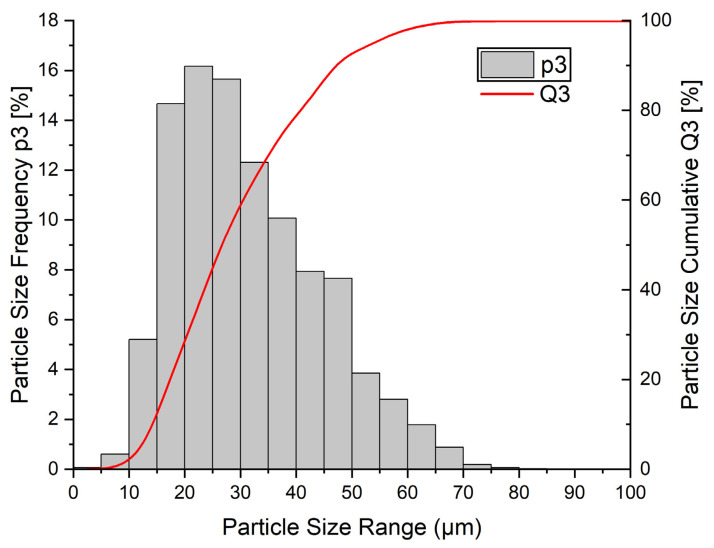
Particle size distribution of the used powder measured by analysis of high-speed imaging (Camsizer X2).

Within this work, the low-alloyed steel 30CrMoNb5-2 is investigated. The extended chemical composition of the alloy is listed in Table 1. Since the particle size distribution (PSD) is significantly impacting the absorptivity of laser radiation, the powder was characterized by a Camsizer X2 (producer: Microtrac Retsch GmbH), using high-speed imaging. The results are visualized in Figure 1, resulting in the following key parameters. The D10/50/90 values indicate that 10/50/90% of the particles have an even or smaller diameter than the presented value:

- D10 = 6.63 µm;
- D50 = 29.18 µm;
- D90 = 49.75 µm.

**Figure 2:**
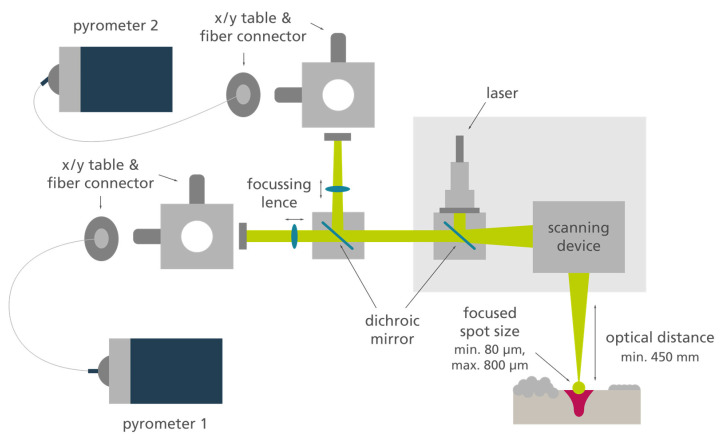
Optical path for the co-axial pyrometry within the used L-PBF system.

All pyrometer investigations were carried out using a Midi+ L-PBF system (producer: Aconity GmbH). Argon was utilized as an inert gas, resulting in an oxygen content of max. 400 ppm. The inert gas flow was set to 1 m/s in order to remove by-products of the welding process. The machine is equipped with four single-mode laser units (wavelength 1070 nm) and corresponding optical units. The optical path of the pyrometers is coaxially coupled into one of these units. This optical unit serves merely as a measurement tool, while the actual laser radiation is guided over a separate unit. The coaxial integration of the pyrometry is shown in Figure 2.

The scanners and the process chamber glass are water-cooled. An additional air cooling based on pressurized air can be activated for both components. According to the machine manufacturer, the setup results in a measurement field of ca. ⌀ 400 µm for the integrated pyrometry. The laser focus diameter can be varied between ca. 80–850 µm. All laser alignments, as well as the pyrometers, have been calibrated according to the manufacturer’s instructions before the measurements. 

Three one-color pyrometers at different wavelengths and one high-speed two-color pyrometer were used. Table 2 shows the minimum and maximum temperature as well as the type, frame rate, linearization status and measured wavelength for each infrared pyrometer. Furthermore, the table shows the notation used in the following graphs. The two non-linearized pyrometers, P3 and P4, can be combined into a two-color pyrometer. Pyrometers P3 and P4 are standard equipment installed by the machine producer, while pyrometers P1 and P2 were integrated by the authors. All data were recorded at 10 kHz using a transient recorder (MF-TransCom-CompaktX-XL). The fitting and data handling were executed in “Origin 2019b”. The EBSD measurements were carried out using a DigiView 5 camera. The step width of the measurements was set to 0.1 µm. 

## 3. Results & Discussion

### 3.1. Realizing Quantitative Measurements by Pyrometry

#### 3.1.1. Validation Setup

**Figure 3:**
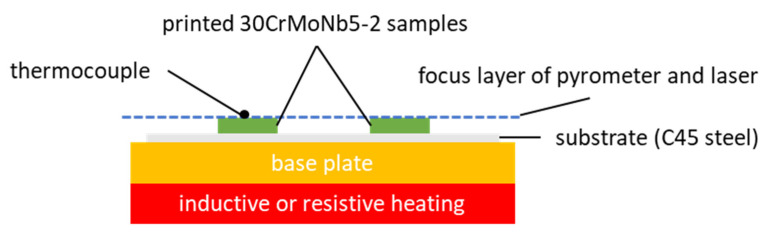
Experimental setup for the interpretation of the pyrometer signals.

Figure 3 shows the experimental setup used to validate the temperature measured by two-color pyrometry as well as to determine the emissivity in dependency of the temperature. For temperatures up to 401 °C, a resistive heating system was used, and an inductive heating system was used for higher temperatures. The heating systems are separated by a base plate from the 2 mm thick substrate (here: C45 steel), which serves as a replaceable build platform. Two 30CrMoNb5-2 cuboid samples of 10 × 10 × 2 mm^3^ are printed onto the substrate plate. A K-Type thermocouple with an accuracy of ±1 °K or ±0.75% is fixed to one of the samples using an M3 stainless steel screw. The thicknesses of the substrate and samples are minimized in order to avoid a loss of temperature between the heating system and the focus layer. When setting the machine to the maximum temperature of 1200 °C (measured inside of the machine close to the heating system), the thermocouple reached a maximum of 938 °C.

**Figure 4:**
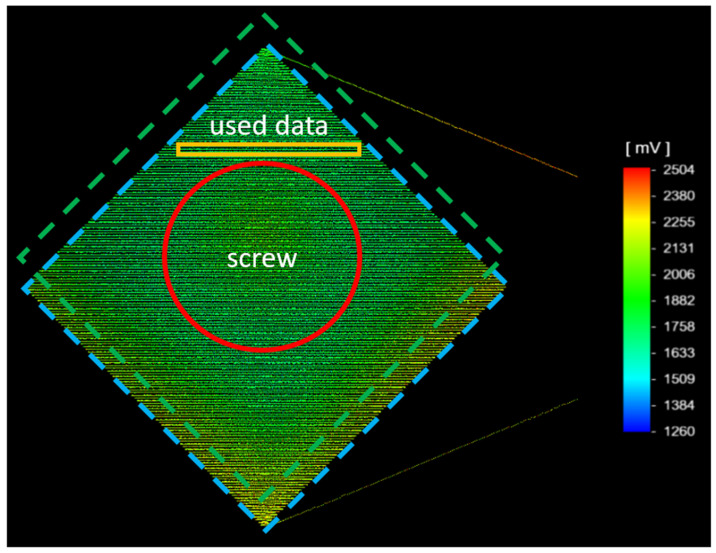
Data recorded while scanning the sample. Scanning field marked in blue. Sample marked in green. Screwhead marked in red. Relevant data used for the following analysis is marked in orange.

After reaching a stable temperature measured by the thermocouple, the samples are scanned by the pyrometers. One exemplary measurement result is visualized in Figure 4. The results show a slight deviation between the scanning field and the actual sample. This is evident by the increased pyrometer signal (yellow and orange signals) on the bottom side. The higher signals result from the radiation of the lower and, therefore, warmer substrate plate. Furthermore, the data recorded on the screwhead show slightly lower temperatures compared to the sample. The rest of the sample exhibits a homogenous signal. For the following investigation, one single scanning track close to the screw head and thermocouple was used in order to avoid tampered data outside the sample or on the screwhead. In general, the sample equipped with a screw and thermocouple shows a slightly lower temperature than the sample without a thermocouple. The difference is more significant for increasing temperatures. Thermocouples and screws seem to result in an increased heat transfer and a lower temperature due to convection. Since only the sample equipped with the thermocouple can be validated, the sample without is not further regarded within this work.

#### 3.1.2. Determining the Emissivity by Linear Regression

**Figure 5:**
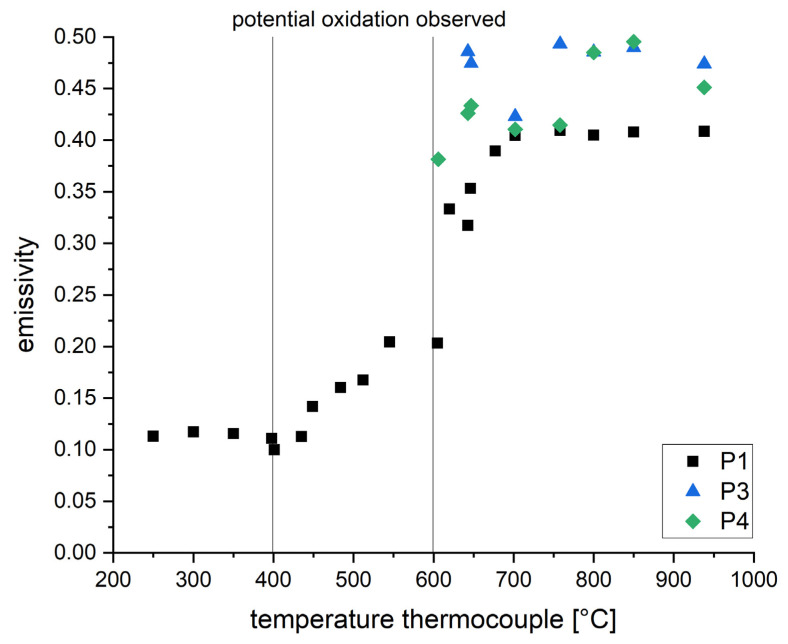
Temperature-dependent emissivity derived by linear regression for the one-color pyrometers P1 (wavelength 1.58–2.00 µm), P3 (wavelength 1.45–1.70 µm) and P4 (wavelength 2.00–2.20 µm).

In order to determine the emissivity by linear regression, measurements were carried out several times at each temperature while setting the emissivity value of the one-color pyrometers to levels between 0.1 and 1 (step size: 0.1). As long as the resulting temperature signal is lower than the temperature obtained by the thermocouple, the emissivity is set too high. The actual emissivity is determined by linear regression between the last measurement resulting in a too-low temperature and the following resulting in a too-high temperature. The resulting emissivities for all investigated temperatures are plotted in Figure 5.

Up to ~435 °C, the emissivity seems to be stable around an average of ~0.12, followed by a linear increase up to ~0.41. During this increase, a change in the color of the samples was observed. Furthermore, the oxygen level within the built chamber decreased from 400–600 ppm to around 150–300 ppm. It is, therefore, possible that oxides impacting the emissivity start to form at the surface of the samples. Between 646 °C and the highest measured value of 938 °C, the emissivity is constant around ~0.41. Regarding the non-linearized pyrometers, the determined emissivity within this last section is slightly higher (0.4–0.5). One aspect of this could be assigned to the fact that all three pyrometers measure different ranges of wavelength. Since the scanning device is not achromatically optimized, there could be optical aberrations generating an error in the measurement.

**Figure 6:**
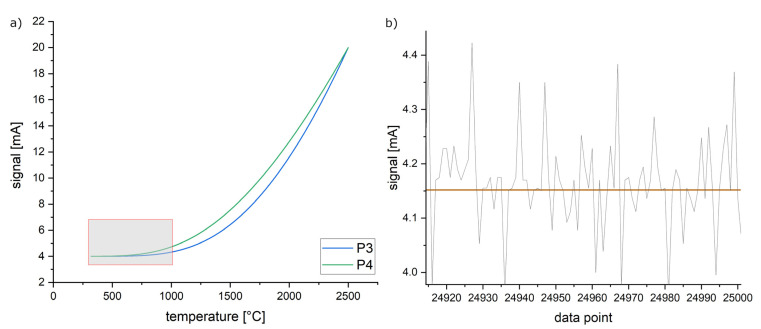
(**a**) Visualization of the manufacturer’s calibration tables regarding the non-linearized pyrometers P3 and P4. Temperatures up to 1000 °C result in a low signal response (marked in red). (**b**) Signal obtained at approx. 700 °C shows pronounced noise, which results in a low signal-to-noise ratio (SNR). Red line represents the mean value used to correlate the corresponding temperature.

Thombansen et al. [21] describe the challenges of L-PBF processing in combination with a coaxially coupled process observation. Since optical elements such as f-theta lenses are monochromatically designed for the laser wavelength, chromatic aberrations of the thermal radiation could lead to a misinterpretation of temperature signals via pyrometry. In addition, the deviation could be caused by measurement noise in combination with the low significance within the calibration table for this temperature range. The calibration tables of P3 and P4 are visualized in Figure 6a. The curves show that temperatures up to 1000 °C result in a low signal of a maximum of ~4.3 mA (P3) or ~4.7 mA (P4). A typical signal, however, shows noise at a range of ±0.2 mA. A precise measurement is, therefore, not possible at these temperatures.

#### 3.1.3. Determining the Emissivity by Comparison to a Theoretical Black Emitter

In general, the emissivity *ε* of a material is the ratio of the specific spectral radiation of a sample *M_S_* and the theoretical specific radiation of the black emitter $M_{∆\lambda ,}$ at the same temperature *T_Ref_* and observed wavelength *λ* (Bernhard, 2014):

(1) $$\varepsilon \left(T_{Ref}\right)=\frac{M_{S}(∆\lambda ,T_{Ref})}{M_{∆\lambda ,}(∆\lambda ,T_{Ref})}$$

Therefore, an alternative approach to identify the emissivity of a surface with a known temperature is to measure the radiation at a set emissivity of 1 (assumption of a black emitter) and compare it to a theoretical black emitter:

(2) $$\varepsilon \left(T\right)=\frac{\int_{\lambda_{1}}^{\lambda_{2}}M_{S}\left(\lambda ,T_{\varepsilon =1}\right)d\lambda }{\int_{\lambda_{1}}^{\lambda_{2}}M_{\Delta \lambda }\left(\lambda ,T_{Ref}\right)d\lambda }=\frac{\int_{\lambda_{1}}^{\lambda_{2}}\frac{2\pi c_{0}^{2}h}{\lambda^{5}\left({\mathrm{e}}^{\frac{hc_{0}}{\lambda kT_{\varepsilon =1}}}-1\right)}d\lambda }{\int_{\lambda_{1}}^{\lambda_{2}}\frac{2\pi c_{0}^{2}h}{\lambda^{5}\left({\mathrm{e}}^{\frac{hc_{0}}{\lambda kT_{Ref}}}-1\right)}d\lambda }$$

*h* = Planck’s quantum of action

*k* = Boltzmann constant

*c*_0_ = light velocity in vacuum

This analytical approach enables the determination of the emissivity based on temperature signals derived from two independent sources (here: thermocouple and pyrometer). However, both integrals are evaluated over the same wavelength range. Therefore, two different black emitter spectra are regarded. Since the observed temperature signals from the thermocouple and pyrometer are close to each other, the absolute errors are assumed to be minor.

**Figure 7:**
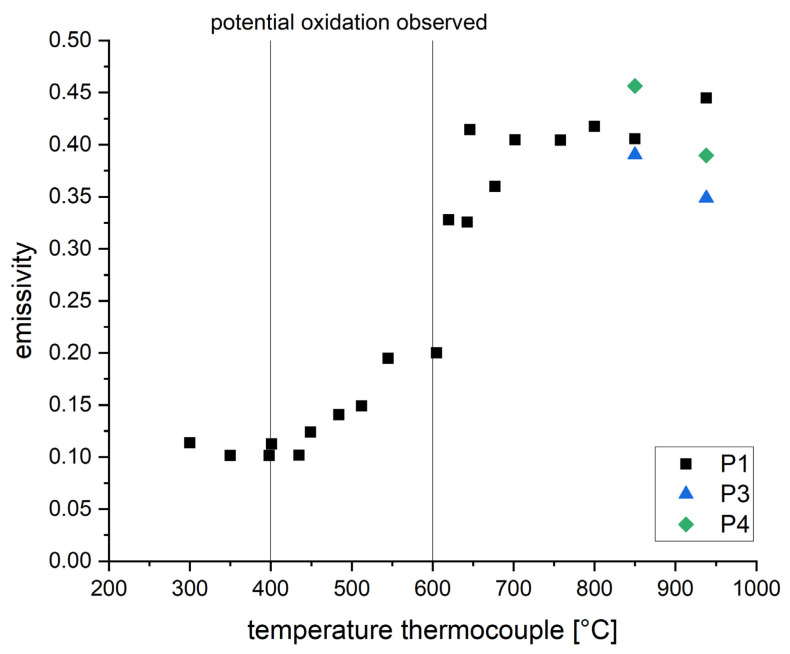
Temperature-dependent emissivity derived by comparison to a theoretical black emitter for the one-color pyrometers P1 (wavelength 1.58–2.00 µm), P3 (wavelength 1.45–1.70 µm) and P4 (wavelength 2.00–2.20 µm).

The emissivity calculated by this approach is shown in Figure 7. The results regarding P1 are in accordance with the alternative approach of linear regression. P3 and P4 show a lower deviation compared to the approach by linear regression. A pyrometer features a minimum error when adjusted to a maximum emissivity. The smaller fluctuations can therefore be associated with the assumed emissivity of 1 (see Formula (2)). In general, the approach presented by Formula (2) could also be used at lower assumed emissivity by considering the theoretical black emitter only partially. This approach has not been regarded since a lower assumed emissivity would also result in an increased error. However, due to the high setting of the emissivity, the resulting pyrometer signals are lower, resulting in the first suitable measurement signal above 850 °C.

#### 3.1.4. Validation of Two-Color Pyrometry

Two-color pyrometry takes advantage of Wien’s approximation when putting Planck’s radiation law at two different wavelengths in the ratio [22]:

(3) $$\frac{I_{1}}{I_{2}}=\frac{\varepsilon_{2}}{\varepsilon_{1}}{\left(\frac{\lambda_{2}}{\lambda_{1}}\right)}^{5}\frac{1-exp\left(\frac{c_{b}}{\lambda_{2}T}\right)}{1-exp\left(\frac{c_{b}}{\lambda_{1}T}\right)}$$

*I* = radiation intensities [Wm^−3^sr^−1^]

*c_b_* = 1.4388 × 10^−2^ m K

And assuming that the emissivity is the same at both wavelengths (Wien’s approximation):

(4) $$\frac{I_{1}}{I_{2}}={\left(\frac{\lambda_{2}}{\lambda_{1}}\right)}^{5}\frac{1-exp\left(\frac{c_{b}}{\lambda_{2}T}\right)}{1-exp\left(\frac{c_{b}}{\lambda_{1}T}\right)}$$

The temperature T can be derived from the ratio of the intensities at different wavelengths:

(5) $$T=\frac{c_{b}(\lambda_{1}-\lambda_{2})}{\lambda_{1}\lambda_{2}}\frac{1}{ln\left(\frac{I_{1}\lambda_{1}^{5}}{I_{2}\lambda_{2}^{5}}\right)}$$

**Figure 8:**
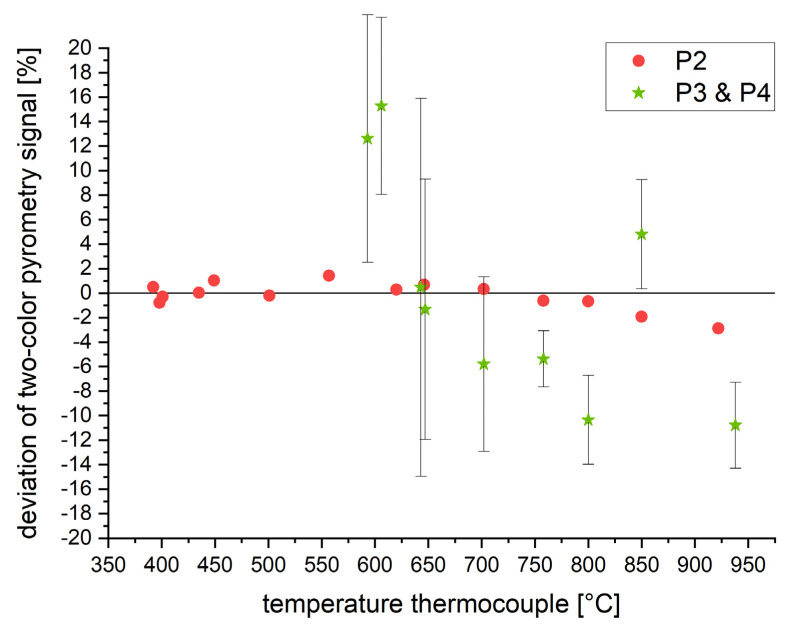
Validating the signals of the two-color pyrometers by comparing the measured temperatures to the signal of the thermocouple. Results are shown for the following pyrometers: P2 (wavelengths 1.45–1.65 and 1.65–1.80 µm) and the combination of P3 and P4 (wavelengths 1.45–1.70 and 2.00–2.20 µm).

However, Wien’s approximation can cause certain measurement errors for certain materials, wavelengths, etc. Therefore, the two-color pyrometry approach is validated for the presented case. Figure 8 shows the deviation of the temperature obtained by two-color pyrometry for P2 and the combination of P3 and P4, compared to the temperature measured by the thermocouple. P2 shows a low deviation of ±2%.

The signals of P3 and P4 are combined by the formulas above. This is done for all measurements, which are executed at different emissivity levels between 0.1 and 1 (step size: 0.1). The results exhibit a higher absolute variation as well as significant standard deviations of the single measurements. Both could result from the investigated temperatures below 1000 °C, resulting in signals distorted by the measurement noise (see Figure 6).

### 3.2. Approach to Measure Cooling Duration

#### 3.2.1. Experimental Setup

**Figure 9:**
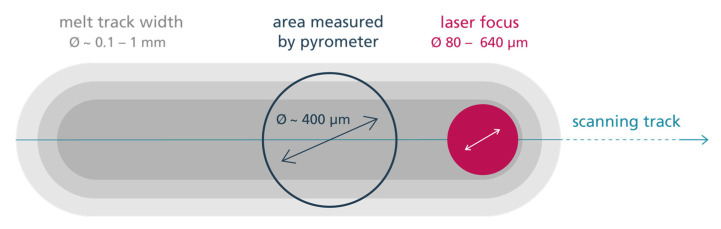
Measuring arrangement in order to characterize a single laser track of specific laser power and scanning speed.

Figure 9 illustrates the measurement setup used in order to investigate the cooling rates or, more precisely, the cooling durations within this work. While the area observed by the pyrometer is resting statically in one position, a single laser track of defined laser power, scanning speed and focus diameter is passing through the field of measurement. The measurements are carried out on 30CrMoNb5-2 sample cubes manufactured by L-PBF measuring 50 × 50 × 10 mm^3^. No powder layer is applied. In order to avoid any influence of inertia effects (e.g., starting movement of mirrors), the laser tracks measure a distance of 50 mm. The pyrometer is placed in the middle of this track. Each track is placed in a new position after the execution of an experiment in order to avoid the impacts of already exposed surfaces. Due to the noise effects regarding P3 and P4 observed in the validation process, the following investigations are conducted using P1 and P2. The emissivity of P1 is set to 0.12. Therefore, measurements above 435 °C will have to be adjusted based on the emissivity plotted in Figure 5 or Figure 7.

It is known that the resulting melt track can be smaller than the area measured by the pyrometer. Regarding the one-color pyrometry, this can lead to a reduced signal since the average radiation within the field is being measured. The two-color pyrometry, however, should not be affected since only the ratio of the measured intensities at different wavelengths defines the measured temperature (see Formula (5)). Therefore, the maximum temperature within the area of measurement is given by the signal. According to Book [23], this is true as long as the maximum temperature is spreading over min. 20% of the measured area.

In order to ensure that the field of measurement is in alignment with the laser track, the XY-tables responsible for the positioning of the field of measurement (see Figure 2) have been moved until reaching the maximum signal within a single laser track experiment. This has been done additionally with the calibration according to the manufacturer’s instructions.

#### 3.2.2. Characteristics of Obtained Signals

**Figure 10:**
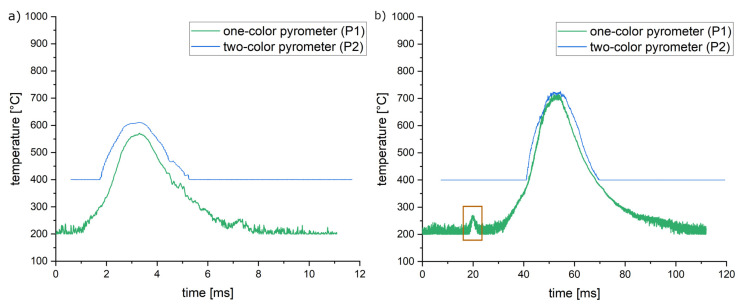
Obtained temperature signals of laser tracks showing typical melt pool by-products during exposure (e.g., smoke, welding beads, sparks). (**a**) Lower energy density: 400 W laser power, 2000 mm/s scanning speed and 80 µm focus diameter. (**b**) Highest investigated energy density: 900 W laser power, 250 mm/s scanning speed and 80 µm focus diameter. Brown mark: Potential welding bead flying through the measurement field of the pyrometer.

Figure 10 shows characteristic signals obtained by the setup above. Measurements resulting in a smaller melt pool showed increased deviation between the one- and two-color pyrometers (for example, see Figure 10a). This can be explained by the fact that the measured area is bigger than the melt pool, resulting in a reduced signal. Bigger melt pools exhibit a smaller deviation (for example, see Figure 10b).

For some measurements, small signal peaks close to the actual peak were observed (see the brown mark in Figure 10b). This can be observed more often with increasing energy densities resulting in more by-products of the melt pool. It is therefore suspected that the peaks present welding beads flying through the field of measurement shortly before or after the passing of the actual laser focus.

In general, the signals show an asymmetric peak with a longer slope on the cooling side. The form of the peak is unexpected and does not correspond to simulation results, as shown by Karayagiz et al. [24,25]. An almost immediate increase followed by an exponential decline resulting in a sharp peak should be expected. Furthermore, the maximum temperature does not correlate with the melting temperature of the material, even though melt tracks are observed. The maximum temperatures increase with the applied energy density. This correlation, however, seems to be linear only in the beginning while converging around 700–750 °C when investigating a laser focus size of 80 µm. Several potential reasons for these unexpected signals are discussed in the following:

1. **Field of measurement not in line with melt track**

This option is ruled out since the maximum signal was obtained during the calibration process. Furthermore, there should be a pronounced deviation between one- and two-color pyrometry in all measurements (e.g., not the case in Figure 10b).

2. **Field of measurement is bigger than melt track**

This option is ruled out since parameter combinations causing melt pools close to 1 mm were investigated, resulting in the same type of signal. Furthermore, the use of two-color pyrometry should rule out this problem.

3. **Optical components warm up and distort signal**

This could have an impact. However, considering the short exposure time, the observed reduction of the temperature seems too high for this effect.

4. **By-products block the radiation from the melt pool**

This seems to be the most probable hypothesis. By-products were visible for almost all measurements. It would also explain why the temperature is converging with increasing energy densities. By-products rising from the melt pool would rapidly cool and start spreading after a short distance. Therefore, the resulting by-product plume is limited in its height and outside temperature. This effect should be more pronounced when exposing a powder layer since loose particles cause more by-products.

**Figure 11:**
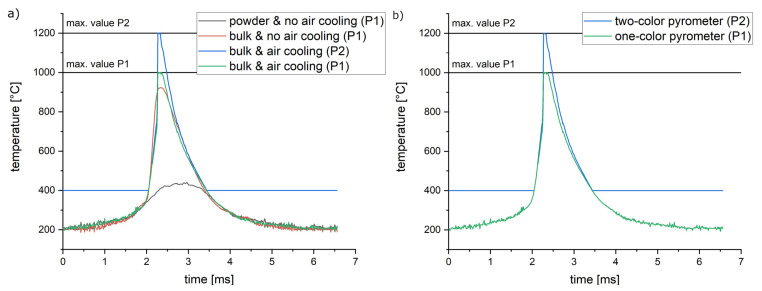
Comparing the impact of by-products and air-cooled optical components onto the temperature signal. Used laser track parameters: 400 W laser power, 1000 mm/s scanning speed and 640 µm focus diameter. (**a**) Comparing the exposure of a single 30 µm powder layer, using only water cooling (P1, black), the exposure of bulk material without air cooling (P1, red) and the P1 and P2 signals at the exposure of bulk material at additional air cooling of the optical components (green and blue). (**b**) Direct comparison of P1 and P2 at improved conditions (bulk material and air cooling).

In order to check hypotheses three and four, process parameters resulting in no visible by-products when exposing bulk material but causing a plume of by-products when exposing a single powder layer are selected. A laser power of 400 W, a scanning speed of 1000 mm/s and a laser focus size of 640 µm are favorable for this verification approach. The parameters were applied under the following conditions:

1. Exposing a **powder layer** of 30 µm with bulk material below and **no air** cooling of the optical units. The black curve in Figure 11a.
2. Exposing **bulk material** directly **without air cooling** of the optical units. The red curve in Figure 11a.
3. Exposing **bulk material** directly and applying **additional air cooling** to the scanner unit and process chamber glass (set at 1.5 bars). Green (P1) and blue (P2) curves in Figure 11a.

The resulting temperature curves in Figure 11a show a strong impact on the by-products resulting from condition one (by-products caused by the powder layer). In addition, the use of additional air cooling of the optical units increases the maximum temperature. Using both optimized measurement conditions (no visible by-products and additional air cooling), the maximum temperatures of P1 and P2 are reached. Above 500 °C, Figure 11b also illustrates a small mismatch between the signals of P1 and P2. This deviation increases with the temperature and could be due to an increasing emissivity, as shown in Section 3.

The curves under optimized conditions exhibit the expectable behavior according to the literature (Karayagiz et al. [24,25]), showing a steep, rising slope followed by an exponential decline. Therefore, both hypotheses three and four prove to have an impact. However, by-products can only be avoided for a small amount of mainly irrelevant process parameter combinations. A methodology for signal processing of measurements distorted by by-products is presented in the following chapter.

#### 3.2.3. Signal Processing for Systematic Investigations

**Figure 12:**
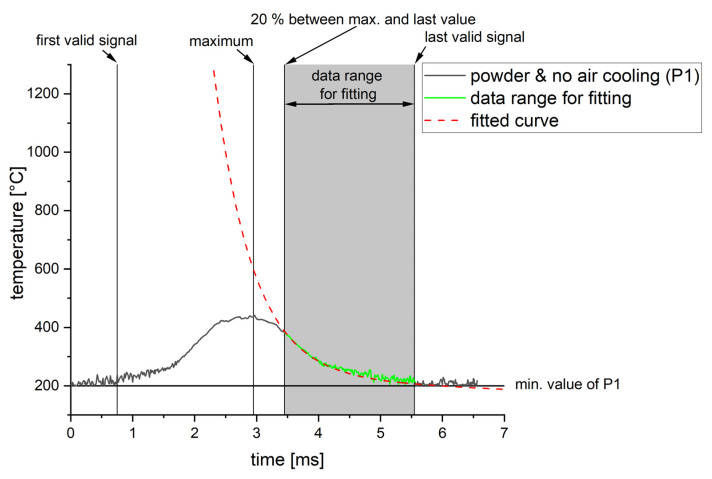
Definition of the data range used for fitting.

The measurements in Figure 11 show that mainly the peak and the increase are distorted due to by-products. The declining slope, however, overlaps with the optimized signals after ~20% between the peak and the last valid signal. Therefore, the declining slope representing the cooling of the melt pool can be seen as valid and used to fit an analytical function (see Figure 12). It can be expected that the observed range of ~20% will only be valid for the regarded case since the size, speed and amount of by-products depend on the chosen process parameters and used material, as shown by Li et al. [26]. The presented validation experiment will therefore be necessary for each material or change in process parameters.

The melt pool’s heat flow rate consists of conduction, convection and radiation:

(6) $$\dot{Q}=\dot{Q_{\kappa }}+\dot{Q_{\alpha }}+\dot{Q_{\varepsilon }}$$

(7) $$mc\frac{dT}{dt}=\kappa \frac{A_{1}}{d}(T_{M}-T)+\alpha A_{1}(T_{M}-T)+\varepsilon kA_{2}(T_{M}^{4}-T^{4})$$

*A*_1_ = surface between melt pool and bulk material

*A*_2_ = surface between melt pool and inert gas flow

*κ* = thermal conductivity

α = heat-transfer coefficient

The ambient temperature *T* is considered to be 0 °C. When replacing all constants with $C_{1}$, $C_{2}$, $C_{3}$ and $C_{4}$:

(8) $$C_{1}\frac{dT_{M}}{dt}=C_{2}T_{M}+C_{3}T_{M}+C_{4}T_{M}^{4}$$

(9) $$\int 1dt=\int \frac{C_{1}}{C_{2}T_{M}+C_{3}T_{M}+C_{4}T_{M}^{4}}dT$$

And considering that $C_{1}$, $C_{2}$, $C_{3}$, $C_{4}$ and $T_{M}$ are positive, the temperature of the melt pool $T_{M}$ can be described by:

(10) $$T_{M}=\frac{\sqrt[{3}]{-C_{2}-C_{3}}}{\sqrt[{3}]{C_{4}*-e^{-\frac{3Ct(C_{2}+C_{3})}{C_{1}}}}}$$

**Figure 13:**
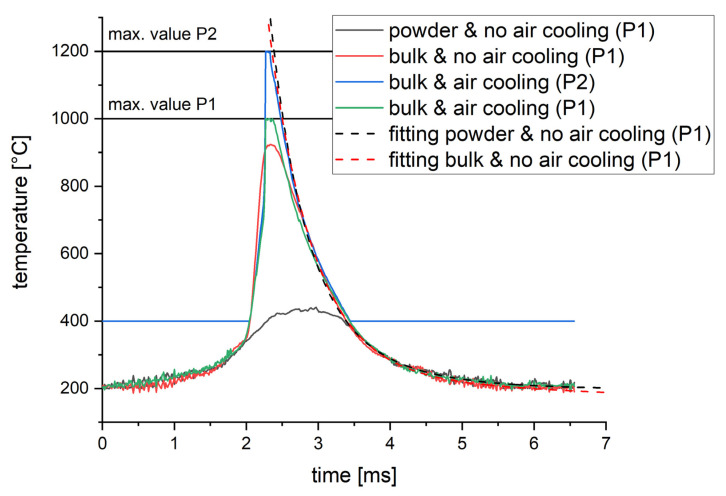
Comparison of the fitted curves and original temperature signals.

When fitting this function to the data obtained by condition one (powder layer and no air cooling) and two (bulk material and no air cooling) within the range defined in Figure 12, the fitting curves in Figure 13 are obtained. Both fitting curves agree with the P2 signal under optimized conditions. The fitting curve for condition one (powder layer and no air cooling) shows no significant deviation. Therefore, the impact of a 30 µm thick powder layer on the cooling rate seems neglectable for the investigated parameter combinations. The presented approach is therefore used in all the following results.

### 3.3. Measurements

#### 3.3.1. Cooling Durations

**Figure 14:**
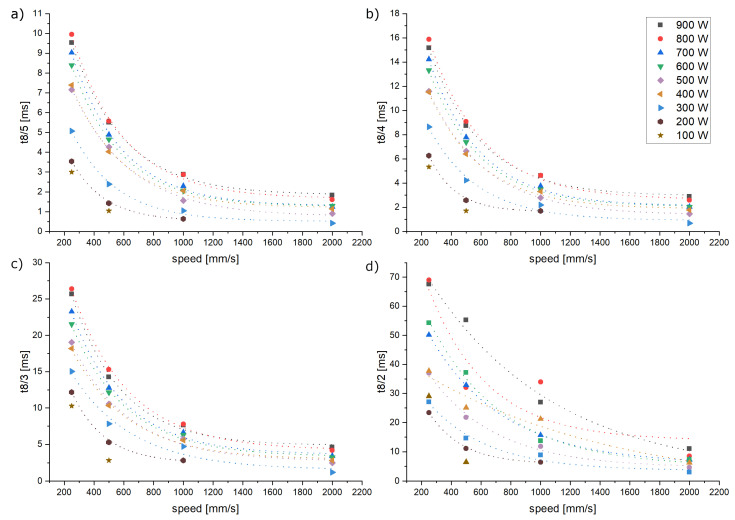
Derived cooling durations representing the cooling rates at a focus diameter of 80 µm. (**a**) 800 °C to 500 °C. (**b**) 800 °C to 400 °C. (**c**) 800 °C to 300 °C. (**d**) 800 °C to 200 °C.

The fact that the cooling rate [°C/s] presents a current value constantly changing over time (see different definitions of literature values in state of the art) makes it a rather unhandy parameter in order to predict microstructural evolutions. Therefore, the cooling duration between the austenitization temperature of around ~800 °C and typical values for the phase transformation to martensite and bainite are calculated based on the fitted curves. The resulting cooling duration between 800 °C and 500 °C (t8/5), 400 °C (t8/4), 300 °C (t8/3), as well as 200 °C (t8/2) are summarized in Figure 14. Cooling durations between ca. 0.5 and 70 ms are observed. The scanning speed has an exponential impact, while the laser power shows a linear effect on the cooling duration. Based on the coefficient of determination R^2^ obtained by linear regressions, the linear correlation is especially significant for slower exposure speeds. At 2000 mm/s R^2^ between 0.31 and 0.76, at 1000 mm/s R^2^ between 0.62 and 0.92, at 500 mm/s R^2^ between 0.86 and 0.93 and at 250 mm/s R^2^ between 0.89 and 0.98 were observed. The reason for this could be unstable melt pools (balling phenomena) which occur and have been observed at higher exposure speeds. This would result in increased deviations within the measurements. Furthermore, the linear (impact of laser power) as well as the exponential regression (impact of exposure speed) show a higher significance for the cooling duration t8/5, t8/4 and t8/3 for t8/2. This could be due to the imprecision of the fitting process.

**Figure 15:**
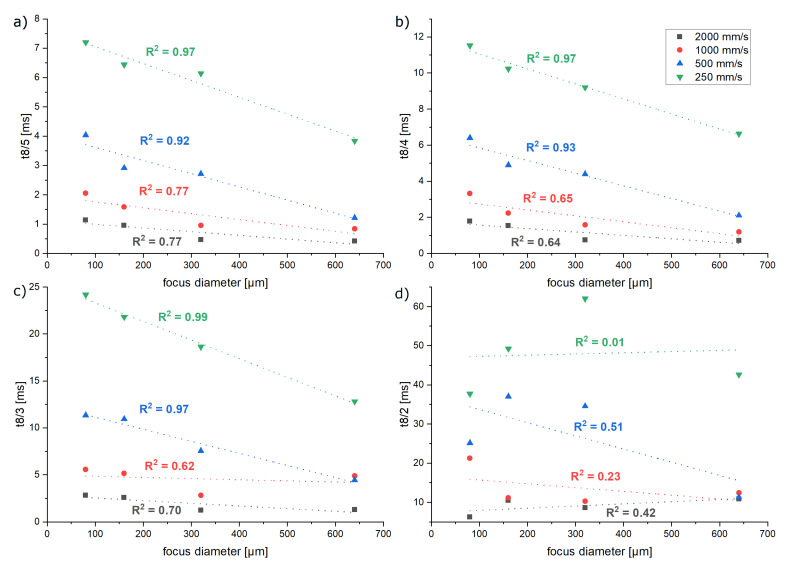
Impact of the focus diameter on the cooling durations. (**a**) 800 °C to 500 °C. (**b**) 800 °C to 400 °C. (**c**) 800 °C to 300 °C. (**d**) 800 °C to 200 °C.

The impact of a changing laser focus diameter is shown in Figure 15. An increasing laser focus diameter results in a reduced cooling duration. One reason for this could be the changing geometry of the melt pool. The energy density decreases with increasing diameter. Therefore, melt pools will be wider and less deep, increasing the surface volume ratio and therefore increasing the heat flows. The impact of the laser focus diameter on the cooling duration is linear. Again, the significance reduces with increasing exposure speed, which could be caused by melt pool instabilities (see R^2^ in Figure 15).

**Figure 16:**
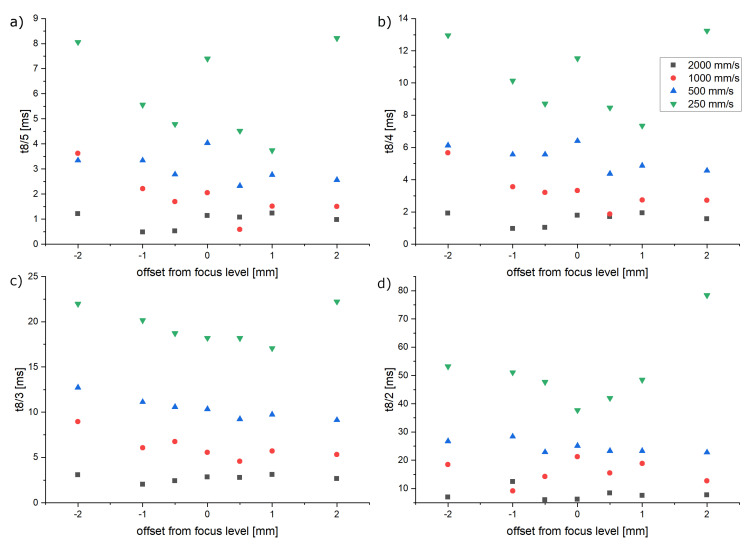
Impact of the focus level offset on the cooling durations. (**a**) 800 °C to 500 °C. (**b**) 800 °C to 400 °C. (**c**) 800 °C to 300 °C. (**d**) 800 °C to 200 °C.

A typical problem when setting up an L-PBF system for a print job is to position the coating system as precisely as possible within the laser focus layer. In order to investigate the impact of such an imprecision, the cooling durations were determined while giving the laser focus level an offset of up to 2 mm. The results (see Figure 16) do not show any significant correlation.

#### 3.3.2. Microstructural Analysis

In order to investigate the impact of varying cooling duration on the resulting microstructures, EBSD measurements are carried out. This is done for single laser tracks in two different states:

1. **No heat treatment applied (no HT):**
  The laser tracks are placed within the final process layer. No following process layers are exposed.
2. **Heat treatment applied (HT):**
  The laser tracks are placed within the sample geometry. The following process layers are exposed by standard parameters. The exposure of each further layer results in an in-situ heat treatment of the single laser track.

**Figure 17:**
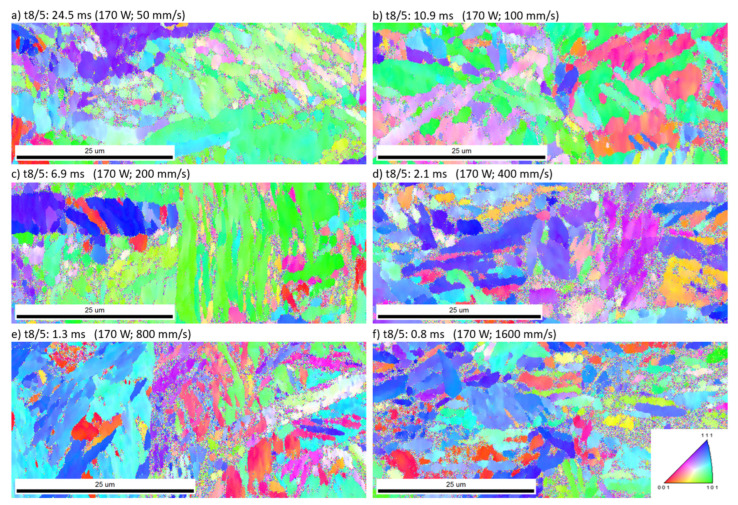
EBSD measurements at non-heat-treated single laser tracks (laser tracks within the last process layer). The cooling duration t8/5 is varied by increasing exposure speeds and a constant laser power of 170 W. (**a**) 24.5 ms (50 mm/s); (**b**) 10.9 ms (100 mm/s); (**c**) 6.9 ms (200 mm/s); (**d**) 2.1 ms (400 mm/s); (**e**) 1.3 ms (800 mm/s); (**f**) 0.8 ms (1600 mm/s).

Investigations by light microscopy on etched and non-etched samples did not show any significant characteristics. The EBSD results of the non-heat-treated single laser tracks are shown in Figure 17. All measurements for heat-treated and non-heat-treated single laser tracks reveal noise between areas of similar crystal orientation. The same phenomenon is observed within L-PBF bulk material. One reason for such a phenomenon could be an insufficient sample preparation resulting in surface-defects or -contamination. In order to rule out this problem, a sample manufactured by hot-rolling has been processed in the same way. No relevant amount of noise has been observed within this measurement. It is therefore concluded that the measured noise either represents areas of severe deformation or potential amorphization due to extremely high cooling rates. Luo et al. [27], for example, show similar EBSD results for amorphous metals manufactured by L-PBF. A higher resolution within the EBSD measurement could help to identify areas of severe deformation. However, a measurement of sufficient resolution was not successful yet.

**Figure 18:**
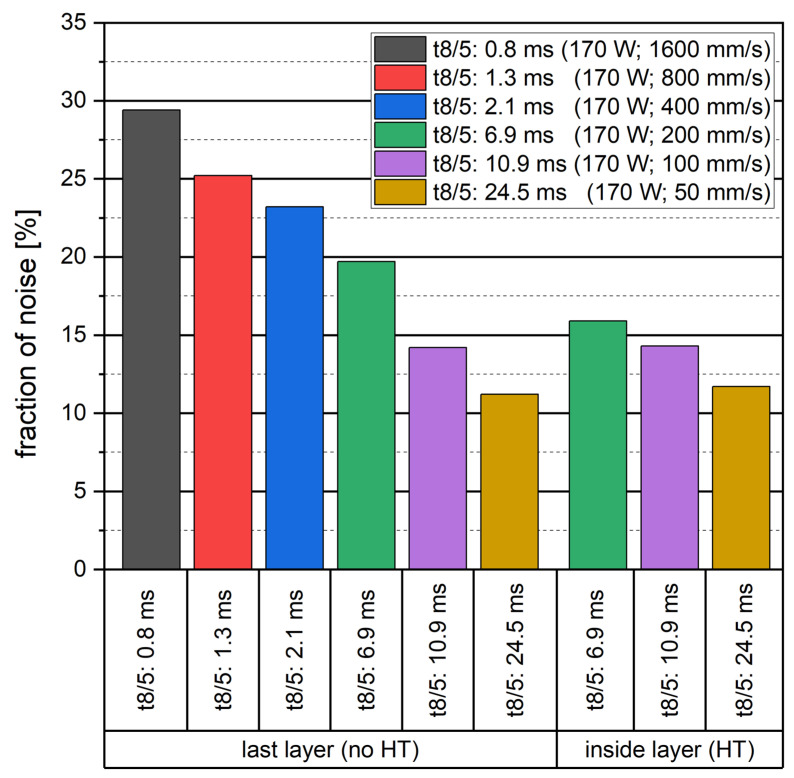
Correlation of cooling duration and fraction of noise within EBSD measurements of heat-treated (HT) and non-heat-treated (no HT) single laser tracks. Value is presenting the ratio of areas that are assumed to present areas of extreme deformations or potential amorphization due to high cooling rates.

When comparing the fraction of noise within the single laser tracks, a clear decreasing trend with increasing cooling duration is visible. Figure 18 shows the correlation between the cooling duration t8/5 and the resulting ratio of noise. This trend is in accordance with the assumption of microstructures resulting from severe deformations or amorphous areas. While the in-situ heat treatment of the laser tracks does not show any significant effect on the fraction of noise regarding slower cooling durations of 24.5 ms and 10.9 ms, the laser track resulting from a cooling duration of 6.9 ms shows a significant decrease in noise. This could be due to annealing effects.

**Figure 19:**
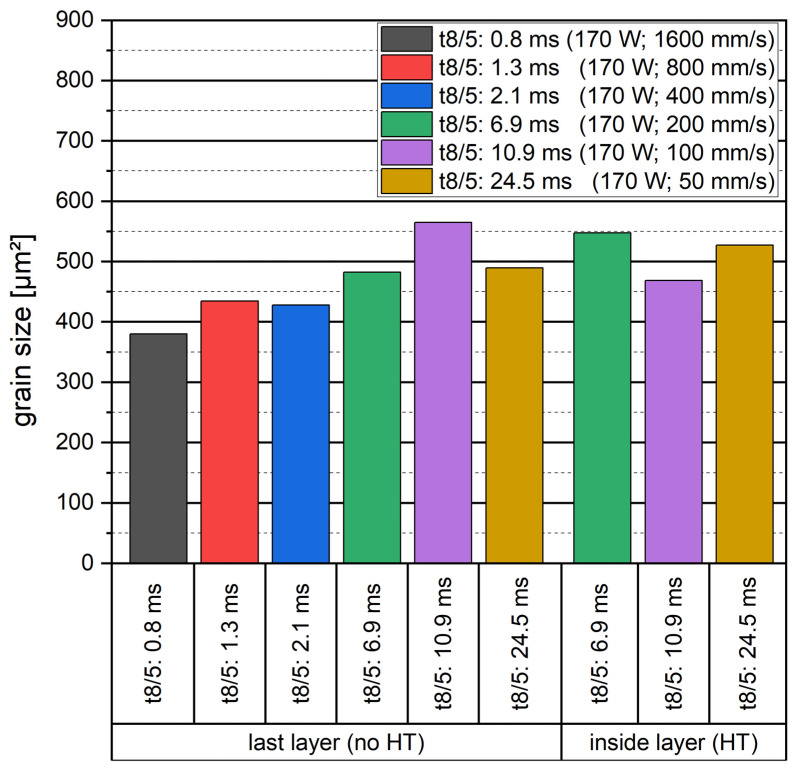
Correlation of cooling duration and grain size measured by EBSD for heat-treated (HT) and non-heat-treated (no HT) single laser tracks.

It is well known that the grain size within a microstructure will increase with the cooling duration. Therefore, Figure 19 presents the average grain size in µm^2^ for the investigated cooling durations. The results show a tendency to increase grain sizes. The in-situ heat treatment does not show any significant impact on the grain size.

## 4. Conclusions

The findings of this work prove that the cooling rate in L-PBF can be measured by pyrometry. A novel approach is introduced in order to overcome the observed problem of signal disturbing by-products within the measurement zone. The four following main topics have been addressed in order to realize the first systematic investigations regarding the correlation of exposure parameters, cooling rates and resulting microstructure:

1. **Quantitative measurements by pyrometry within L-PBF have been realized:**
  Two different approaches have been introduced in order to correlate the pyrometer signal to a temperature-dependent emissivity within the L-PBF system. The material’s emissivity has been measured up to 938 °C (see Figure 5 and Figure 7). These data can serve as a calibration table for future measurements by one-color pyrometry. Furthermore, the use of two-color pyrometry has been introduced, showing a maximum deviation of ±2%.
2. **Introducing a novel approach in order to measure cooling durations of single laser tracks:**
  Within the single laser tracks experiments, by-products have been identified as the main source of error in measuring the cooling behavior. In fact, measurement within the actual melt pool is not possible. A newly developed and validated fitting method has been introduced, enabling statements about the cooling rates based on incomplete measurement data due to by-products.
3. **Systematic investigation of cooling duration**
  Based on the approach above, systematic investigations regarding the cooling duration have been carried out. The results show the correlation between cooling duration and process parameters such as laser power, scanning speed, laser focus size and laser focus offset. The gained quantitative information can be used for simulation validation as well as the understanding of microstructural evolution and can therefore support the future development of FGMs.
4. **Observed phenomena in microstructure**
  EBSD measurements on single laser tracks reveal wide areas of noise. It is concluded that these areas represent either severe deformation or potential amorphization due to extremely high cooling rates. The fraction of noise within the laser tracks correlates with the cooling duration and therefore supports the hypothesis above.

The results support the validation of simulation results as well as the development and optimization of exposure parameters and resulting mechanical properties. The knowledge gained about the cooling duration supports the understanding of microstructural formation mechanisms. In addition, this work presents the foundation for future two-dimensional pyrometry experiments. These would support a deeper understanding of annealing effects within the L-PBF process, which result from the exposure of the following process layers. The results of this work also promote the use of pyrometry as a tool for process monitoring.

## Figures and Tables

**Table 1:** Chemical composition of the used alloy in weight percent.

Element	C	Cr	Mn	Ni	Mo	Si	Cu	Al
wt. pct	0.29	0.91	0.73	0.33	0.27	0.26	0.05	0.04

**Table 2:** Used IR pyrometry devices within this work and notations used in the following results.

	Kleiber KGA 740-LO	METIS H322	Kleiber KGA 740-LO	Kleiber KGA 740-LO
Notation	P1	P2	P3	P4
Type	one-color	two-color	one-color	one-color
Frame rate	100 kHz	25 kHz	100 kHz	100 kHz
Linearization	yes	yes	no	no
Tmin [°C]	200	400	500	500
Tmax [°C]	1000	1200	2500	2500
Wavelength [µm]	1.58–2.00	1.65–1.80 1.45–1.65	1.45–1.70	2.00–2.20

## Data Availability

Data will be made available upon request.
